# Tree Size Inequality Reduces Forest Productivity: An Analysis Combining Inventory Data for Ten European Species and a Light Competition Model

**DOI:** 10.1371/journal.pone.0151852

**Published:** 2016-03-21

**Authors:** Thomas Bourdier, Thomas Cordonnier, Georges Kunstler, Christian Piedallu, Guillaume Lagarrigues, Benoit Courbaud

**Affiliations:** 1 Irstea, UR EMGR, Centre de Grenoble, 2 rue de la Papeterie-BP 76, F-38402 Saint-Martin-d’Hères, France; 2 Univ. Grenoble Alpes, F-38402 Grenoble, France; 3 AgroParisTech, UMR1092, Laboratoire d’Étude des Ressources Forêt-Bois (LERFoB), ENGREF, 14 rue Girardet, CS14216, FR- 54042 Nancy Cedex, France; Technical University in Zvolen, SLOVAKIA

## Abstract

Plant structural diversity is usually considered as beneficial for ecosystem functioning. For instance, numerous studies have reported positive species diversity-productivity relationships in plant communities. However, other aspects of structural diversity such as individual size inequality have been far less investigated. In forests, tree size inequality impacts directly tree growth and asymmetric competition, but consequences on forest productivity are still indeterminate. In addition, the effect of tree size inequality on productivity is likely to vary with species shade-tolerance, a key ecological characteristic controlling asymmetric competition and light resource acquisition. Using plot data from the French National Geographic Agency, we studied the response of stand productivity to size inequality for ten forest species differing in shade tolerance. We fitted a basal area stand production model that included abiotic factors, stand density, stand development stage and a tree size inequality index. Then, using a forest dynamics model we explored whether mechanisms of light interception and light use efficiency could explain the tree size inequality effect observed for three of the ten species studied. Size inequality negatively affected basal area increment for seven out of the ten species investigated. However, this effect was not related to the shade tolerance of these species. According to the model simulations, the negative tree size inequality effect could result both from reduced total stand light interception and reduced light use efficiency. Our results demonstrate that negative relationships between size inequality and productivity may be the rule in tree populations. The lack of effect of shade tolerance indicates compensatory mechanisms between effect on light availability and response to light availability. Such a pattern deserves further investigations for mixed forests where complementarity effects between species are involved. When studying the effect of structural diversity on ecosystem productivity, tree size inequality is a major facet that should be taken into account.

## Introduction

Over the last few decades, the influence of structural diversity of plant communities on the functioning of ecosystems has been studied for the most part through the lens of species diversity [1–4]. The results indicate that plant diversity influences ecosystem productivity and stability in grasslands [5] and forests [6–8] through several processes such as complementarity of resource use and sampling effects [1]. However, the effects of other major components of structural diversity such as spatial heterogeneity or tree size inequality [9–11] have not been widely explored.

Size inequality conditions size hierarchy among individual trees [12] and has a great influence on competition processes in a forest stand [13]. Moreover, silvicultural systems produce contrasted stand structures with different size distributions and size inequalities [14,15]. Even-aged stand management strategies reduce size inequalities by regenerating a stand in just a few years and by growing a single cohort of trees, whereas uneven-aged stand strategies increase tree size inequality by regenerating a stand continuously, with young trees growing in small gaps between older trees [16]. Despite these theoretical and applied dimensions, the effects of tree size inequality on productivity at the population or community scales have been little studied explicitly. In mixed forest ecosystems, several authors found positive [17,18] and/or neutral [19] relationships between size diversity or stand structural complexity and productivity. For monospecific stands of ponderosa pine (*Pinus ponderosa*), European beech (*Fagus sylvatica*) and silver fir (*Abies alba*), neutral or negative relationships were highlighted [13,19]. In young eucalyptus plantations, a loss in wood biomass was also observed due to increased size heterogeneity of stand structure [20]. Using a modeling approach, Caspersen *et al*.[21] showed a decrease in wood productivity in stands dominated by few large trees compared to dense stands dominated by small trees. In a coniferous forest, Liang *et al*. [22,23] found for different species a decrease in diameter growth and recruitment or an increase in mortality associated with increased size diversity. These different studies indicate that it is crucial for productivity to disentangle the effects of species diversity and tree size inequality. A first step would be to analyze the effect of tree size inequality in monospecific stands for a larger set of species than the one used in previous studies.

Asymmetric competition [24,25] is an essential mechanism that could influence tree size inequality on stand productivity. Competition for light is considered as the main competition process in forest stands and is characterized by strong asymmetry, i.e., larger trees monopolize light resources more than proportionally to their size [26]. This asymmetry leads to a strong competitive hierarchy between dominant and dominated trees [27]. In addition, tree species vary greatly in terms of shade tolerance, i.e., the capacity for a dominated individual to survive and grow at low light levels, which determines their response to asymmetric competition [28]. Since shade-tolerant species can grow at low light levels, we can therefore hypothesize that their productivity could be less sensitive to tree size inequality than the productivity of shade-intolerant species. Cordonnier & Kunstler [13] showed that in monospecific stands, there was a negative effect of tree size inequality on stand production that increased with the sensitivity of species to asymmetric competition. However, this shade-tolerance effect has not been studied explicitly.

At the stand scale, the response of stand production to light can be broken down into two performance components: light interception efficiency (LIE, quantity of light intercepted per unit of above-gournd biomass) and light use efficiency (LUE, biomass production per unit of light interception), which are both species-dependent [29]. Onoda *et al*.[30] showed that LIE and LUE varied with size, taller individuals having a better LIE but a lower LUE. In addition, Laurans *et al*.[31] found that species with a small stature were more tolerant to light competition. These differences could modulate the effect of tree size inequality on stand production. Indeed, when tree size inequality of the stand increases, the competition intensity decreases for larger individuals, but increases for smaller individuals. The variations of LUE and LIE with size should thus determine if a competition release for larger trees compensates a competition increase for smaller trees and thus translates into a globally positive or negative effect at the stand scale (see Binkley [32] for a discussion of the same processes throughout stand development).

The objective of this study was to clarify the effect of tree size inequality on stand productivity of monospecific forest stands and how this effect may vary between species. Based on a previous study [13], we expected a negative effect of tree size inequality on stand production and a decrease in this effect with species shade tolerance. We studied ten species with contrasted shade tolerance using the forest inventory from the National Geographic Agency (NGA) plot data. We explored the respective role of LIE and LUE in explaining the effect of tree size inequality on productivity with Samsara2, a forest dynamics model using a detailed algorithm of light interception by trees.

## Materials and Methods

### Plot data

We used plot data from the NGA data base distributed on a 1km by 1km grid over France (NGA, http://www.inventaire-forestier.ign.fr) that combines dendrometric and ecological data. Details on the NGA inventory protocols can be found in S1 Appendix. At the tree level, species identity, height, circumference at breast height (c_130_) and 5-year radial increments were available. We used data from 2006 to 2011 representing more than 44,000 plots. We excluded plots in coppice stands, plots where a cut had been applied in the last 5 years, plots with a single tree and plots in plantations. We then selected monospecific plots, defined as being composed of a minimum of 80% of their basal area represented by a single species. Plot basal area is the sum of individual tree basal areas, i.e., the cross-section of their trunks at breast height (1.30 m). The stand basal area at the time of measurement was computed based on the circumference of each living tree and their respective weight according to size of the subplot they belong to. We then computed the stand basal area five years before using the following available items: (i) the five years radial increment of living trees, (ii) trees that died during the period (alive five years ago), (iii) trees that were recruited during the period (their c_130_ were below the minimum circumference five years ago according to their five years radial increment).

We used only monospecific plots of the ten most represented species in this data set. The number of plots ranged from 1142 to 152 depending on the species (Table 1).

**Table 1:** Characteristics of the plots for the ten species studied.

Species (Abb^a^)	Nb plots	G (m^2^)	Dq (m)	Gini^b^ -	Shade tolerance-	WB (mm)	SGDD (°C)
*Pinus sylvestris* (PinSyl)	1142	22.7 (1.1–69.6)	0.22 (0.06–0.62)	0.34 (0–0.75)	1.67	34.3 (0.1–108.1)	1561.3 (589.7–2605.2)
*Quercus petraea* (QuePet)	1082	23.8 (0.8–84.6)	0.28 (0.08–0.78)	0.39 (0.01–0.82)	2.73	49 (1.3–118.7)	2007.4 (916.4–2602.7)
*Quercus robur* (QueRob)	870	20 (1.1–63.9)	0.28 (0.09–0.93)	0.40 (0.01–0.82)	2.45	55.3 (10.1–116.4)	1843.7 (1020.9–2864.6)
*Pinus pinaster* (PinPin)	860	24.7 (0.8–75.3)	0.29 (0.08–0.75)	0.28 (0.02–0.78)	NA	51 (0.2–109.1)	2418.7 (1223.0–3415.4)
*Fagus sylvatica* (FagSyl)	813	28.2 (0.8–106.8)	0.29 (0.08–1.18)	0.39 (0–0.89)	4.56	46.4 (0.3–129.1)	1455.9 (648.9–2667.8)
*Quercus pubescens* (QuePub)	667	17.5 (0.9–65.9)	0.18 (0.08–0.65)	0.32 (0–0.76)	2.31	30 (0.1–94.4)	2135.2 (1215.1–3300)
*Abies alba* (AbiAlb)	421	36.67 (0.9–90.5)	0.30 (0.08–0.66)	0.45 (0.01–0.78)	4.6	48 (0.1–130)	1260.5 (470.1–2135.2)
*Pinus halepensis* (PinHal)	252	17 (1.1–58.3)	0.23 (0.09–0.48)	0.36 (0–0.73)	1.35	29.1 (0.84–95.4)	2729.6 (2041.8–3499.7)
*Picea abies* (PicAbi)	232	35.2 (1.1–86.3)	0.30 (0.08–0.66)	0.40 (0–0.75)	4.45	54.1 (0.5–132.7)	1093.5 (444.6–1938.4)
*Larix decidua* (LarDec)	152	22.5 (1.5–68.3)	0.29 (0.1–0.73)	0.36 (0–0.73)	1.46	37.1 (0.1–90.9)	827.2 (423.2–2415.2)

Mean values are presented in the table with range between parentheses. G: basal area; Dq: quadratic mean diameter; Gini: Gini coefficient; Shade tolerance: based on Niinemetz and Valladares (2006); WB: Water budget; SGDD: sum of growing degree days above 5.56°C.

^a^ Abbreviations used later in the figures for the species names are presented here.

^b^ The value of 0 as minimum for Gini for certain species are due to very few plots (7 in total) composed of only 2 trees with the same basal area.

We calculated basal areas and quadratic mean diameters for each plot from individual tree data. In this study, we defined stand production as the basal area annual increment on the plot, calculated using the following equation adapted from Vallet & Pérot [33]:

$$dG=\frac{1}{5}\sum \begin{array}{l} i \end{array}\frac{10^{-4}}{4\pi }(c_{130,i}^{2}-\left(c_{130,i}-2\pi \times ir_{5,i}{)}^{2}\right)w_{i}$$ (1)

with:

- *dG* (m^2^ha^-1^year^-1^) the mean annual plot basal area increment over 5 years;
- $\frac{1}{5}$ is used to calculate the mean annual plot basal increment as we had growth over a 5 year period and 10^−4^ is used to convert cm^2^ to m^2^,
- *c*_130,*i*_ (cm) the circumference at breast height of tree i,
- *ir*_5,*i*_ (cm) the radial increment over 5 years of the tree i obtained with core data,
- *w*_*i*_ (trees.ha^-1^) the weight of tree i for 1 hectare (taking into account plot border effects).

The NGA protocol changed during the 6 years considered in this study. Since 2009, “simplified trees” have been introduced to reduce field work. Only species and circumferences were recorded on these trees. To predict the radial increment and height of these trees, we used the prediction of the average growth rate of the measured individual of the same species and same size class (S1 Appendix).

To account for climatic variation across the sampling area we calculated two variables that are known to influence growth [34]: the sum of growing degree days (the sum of daily temperatures exceeding 5.56 C, *SGDD*) and the year’s water budget (*WB*). Temperature, precipitation and solar radiation were modelled and mapped for the whole France [35–37]. The water budget was computed using the maximum soil water capacity estimated for each NGA plot using data from a soil pit [38], potential evapotranspiration [39] and precipitation with the Bugmann & Cramer model [40].

### The Gini coefficient index for tree size inequality

Several indices have been used to quantify size inequality or size diversity in forest stands [11,41]. Particularly, and partly due to its popularity for the quantification of species diversity, the Shannon entropy index has received much attention and has been mobilized in many studies [18,22,23,42–44] However, several authors recently advocated that other indices presents better properties and should be preferred. This is the case for the Gini index which is continuous and more directly related to size hierarchy and thus better linked to asymmetric competition between trees [13,45]. In addition, several studies indicated that it performs better in discriminating stands of different diameter distributions [46,47]. It is therefore a good candidate to address the effects of stand inequality on stand productivity. In this study, we applied the Gini index to individual tree basal areas.

The equation to compute the Gini index is [13,15,48]:

$$Gini=2\frac{\sum_{i=1}^{n}ig_{i}}{nG}-\frac{n+1}{n}$$ (2)

where *g*_*i*_ is the basal area of tree *i* (trees are sorted in ascending order), *G* the total basal area and *n* the number of trees.

**Fig 1:**
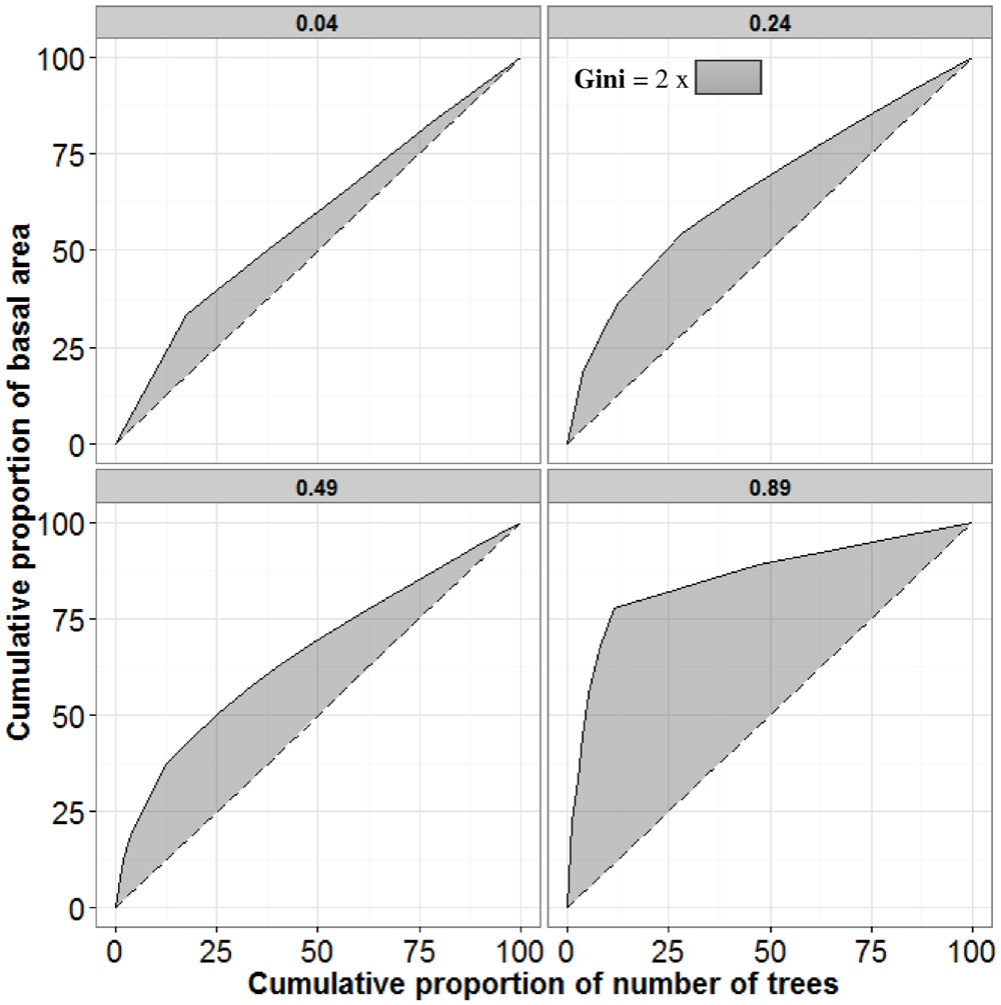
Lorenz curve to calculate the Gini coefficient index for 4 different inventory plots. The Gini coefficient values are presented in the title of each facet. Trees are ranked according to their size (in our case individual tree basal area) in a descending order (according to Valbuena *et al*. 2013 [49]). The dashed lines represent the line of absolute equality.

The Gini coefficient index ranges from 0 (perfect equality, where all values are the same) to 1 (maximum theoretical inequality). In practice the values of Gini indexes observed for the distributions of individual tree basal areas in forest stands of more than 0.25 ha are often between 0.2 and 0.7 [15] (Fig 1).

### Species shade tolerance

To characterize species’ shade tolerance we used two different indexes: Ellenberg’s indicator values and Niinemets & Valladares’ index [50]. Ellenberg’s indicator ranges from 3 to 9 for woody species and was developed in central Europe to characterize species potential to grow in the understory. Niinemets & Valladares’ index ranges from 1 (very intolerant) to 5 (very tolerant) and is available for 806 temperate tree species in North America and Europe.

### Plot productivity analysis

To analyze the effect of tree size inequality on stand production all things being equal, we took into account potential confounding effects caused by the variability of plot structures and environmental conditions in our data set. To this end, we developed a model relating stand production to three stand structural attributes and two environmental variables. We selected total basal area (*G*) to quantify stand density, mean plot quadratic diameter (*Dq*) to quantify development stage and the Gini coefficient index (*Gini*) to quantify tree size inequality. Based on previous studies [6,51] we expected that stand production increases as a convex power function of the plot basal area (an indicator of stand density) and decreases with the plot quadratic mean diameter (an indicator of stand development stage).

Stand production was thus modeled as follows:

$$\text{d}G=\;\alpha \;G^{\beta }\;e^{\gamma Dq}e^{\delta Gini}$$ (3)

where α is a linear combination of site effect components such as:

$$\alpha =e^{\alpha_{0}+\alpha_{1}WB+\alpha_{2}SGDD}$$ (4)

where *WB* and *SGDD* are, respectively, the water budget of the year and the sum of growing degree days above 5.56°C for each plot location.

We quantified the effect of tree size inequality on productivity by the value of parameter δ from Eq 2. A positive value of δ indicates a positive effect of tree size inequality on productivity, and a negative value of δ a negative effect of tree size inequality.

### Samsara2

Samsara2 is an individual-based, spatially explicit forest dynamics simulation model. It has been used to simulate the dynamics of uneven-aged forest stands (combining regeneration, growth and mortality) in mixed and uneven-aged mountain forests [52]. The annual basal area increment of individual trees is modeled as a function of the amount of light they intercept during a growing season. Light interception is calculated in 3D with a ray-tracing algorithm [53]. Each time a ray crosses the crown of a tree, it loses a fixed proportion of the incident energy captured by the tree. The amount of energy intercepted by a tree, during a growing season, in MJ/yr, depends on the size of its crown and on the size and spatial distribution of its neighbors. The relationship between individual tree basal area increment and intercepted energy is modeled as a convex power function. Parameters estimated from field data (unpublished data) are available for two species out of the ten investigated in our study: Norway spruce (*Picea abies*) and silver fir (*Abies alba*). For European beech (*Fagus sylvatica*), some parameters used in Samsara2 were the same as for silver fir because of missing data, but a specific calibration for beech was made for architecture allometries (equations linking height, crown dimensions and aboveground volume to diameter). We used the number of trees per diameter class of each NGA plot to build corresponding initial 1-ha simulation stands (edge effects are managed with a torus). We simulated plot growth over a 10-year period. We calculated plot productivity using two different metrics: first, as the sum of individual tree basal area increments (similar to the NGA) and second, as the sum of individual aboveground volume increments, using the equations developed by Vallet *et al*.[54]. We used the simulated light intercepted by each tree to calculate light interception efficiency (LIE) at the plot scale (ratio between intercepted energy and incident energy above canopy). We then calculated light use efficiency (LUE) as the ratio between plot productivity (basal area increment only) and intercepted energy.

### Statistical analysis

We conducted all statistical analyses using the R open-source software (Version 3.1, R Core Team). To calibrate our production models on NGA data, we performed linear regressions (lm function) using the log-transformed version of the model for each species. The log transformation of growth data has been applied in many studies [55–57] due to classic log-normal distribution of basal area increments and is well suited for multiplicative errors structure, but provide poor fit if the underlying error structure is additive [58]. In our study, the use of the log-transformation is justified as log-transformed models with a multiplicative error (lm function in R) performed better than non-linear ones (nls function in R) (S2 Appendix). With the full model, we tested all variables systematically and kept only variables with a significance level below 5% (*p*-value<0.05). In addition, we compared a model with the Gini index and a model without the Gini index using the Akaike information criterion (AIC) [59] to evaluate strength of support of this variable. We tested its significance with a log-likelihood ratio test between the two nested models. We assessed normality and heteroscedasticity visually using normal Q-Q plots and plots of residuals vs. predicted. We analyzed the data produced by Samsara2 using model (2), but we considered the parameter α as a constant because no site variability was included in Samsara2 (α_1_ = α_2_ = 0). Based on a preliminary exploration of LIE and LUE, we found that the same relationship (Eqs 4 and 5) adequately explained the variation of LIE and LUE (including G, Dq and the Gini index). LUE is the ratio between productivity and the total light interception of a stand (Eq 5).

$$LIE=\;\alpha_{I}\;G^{\beta_{I}}\;e^{\gamma_{I}Dq}e^{\delta_{I}Gini}$$ (5)

$$LUE=\frac{dG}{LIE}=\alpha_{U}\;G^{\beta_{U}}\;e^{\gamma_{U}Dq}e^{\delta_{U}Gini}$$ (6)

We centered and standardized all variables to facilitate the comparison of their effects on productivity, LUE and LIE.

## Results

First, in accordance with our hypothesis, for all species we observed a positive effect of stock (basal area) (0.39 < β < 0.70) and a negative effect of development stage (mean quadratic diameter) (−0.78 < *γ* < −0.37) on productivity.

### Effect of tree size inequality and functional characteristics on stand production

**Fig 2:**
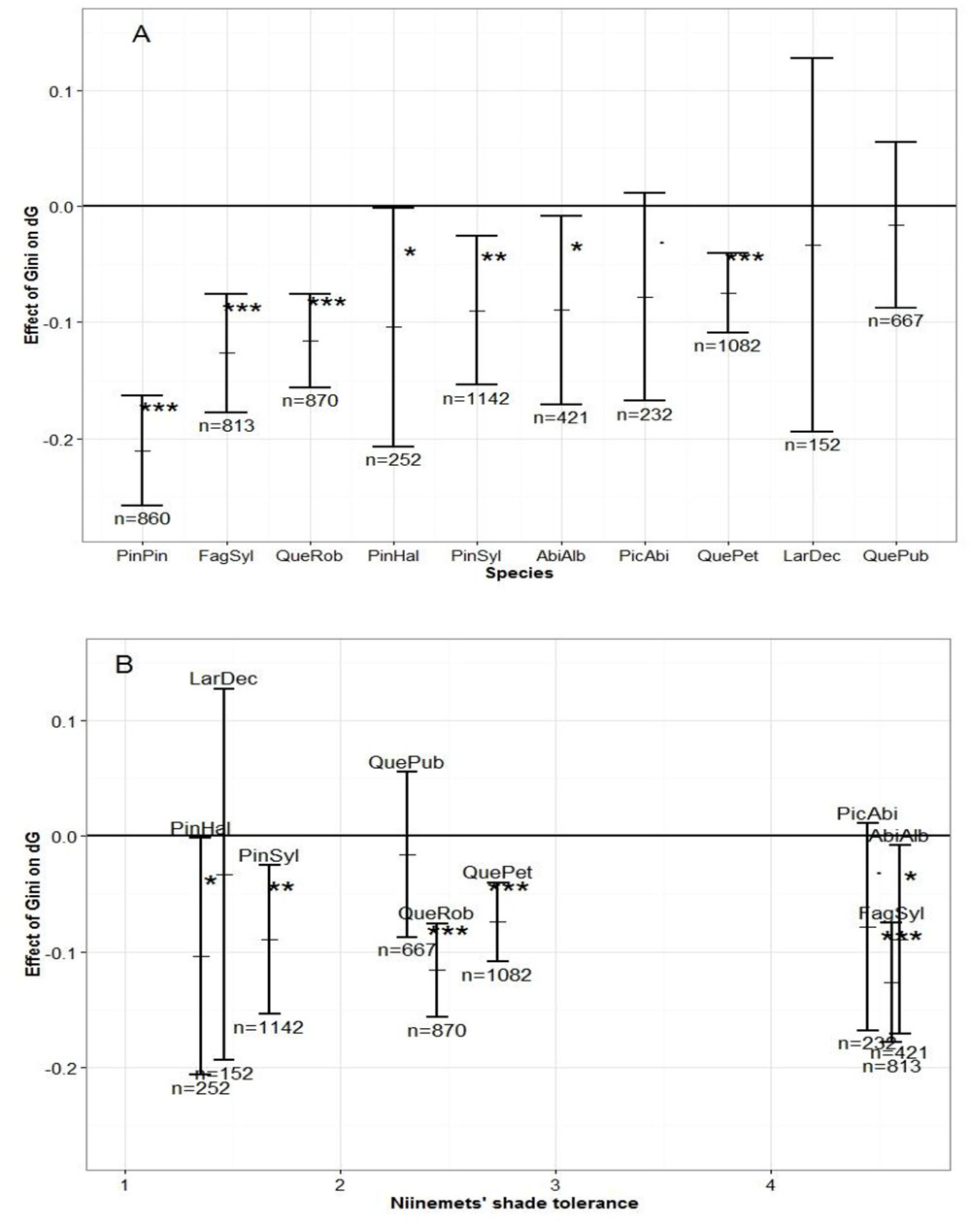
Effect of Gini coefficient (δ from Eq 3) on productivity (dG). The results are presented according to species sensitivity to size heterogeneity (A) or to their shade tolerance (B). *Pinus Pinaster* was removed from (B) because there is no shade tolerance value in Niinemets & Valladeres (2006). Levels of significance are indicated on the graph based on a log-likelihood ratio test: (.) *p*-value <0.1, (*)<0.05, (**)<0.01, (***)<0.001. *p*-values are available in S3 Appendix for all variables.

We found a significant negative effect of the Gini coefficient (parameter δ, see Eq 2; based on a log-likelihood ratio test) indicating a negative effect of tree size inequality on plot productivity, for seven out of the ten species studied (Fig 2a). This was confirmed by an AIC comparison between the models with or without the Gini coefficient (not shown). However, we observed no effect of tree size inequality on the productivity of *Quercus pubescens*, *Larix decidua* and *Picea abies*. At the other extreme, *Pinus pinaster* showed a stronger sensitivity to tree size inequality (δ = −0.21±0.02) than the other species (delta between −0.12 and −0.07 for the six other species). We found no clear effect of shade tolerance indices on the magnitude of parameter δ (Fig 2b).

### Effect of tree size inequality on LUE and LIE

**Fig 3:**
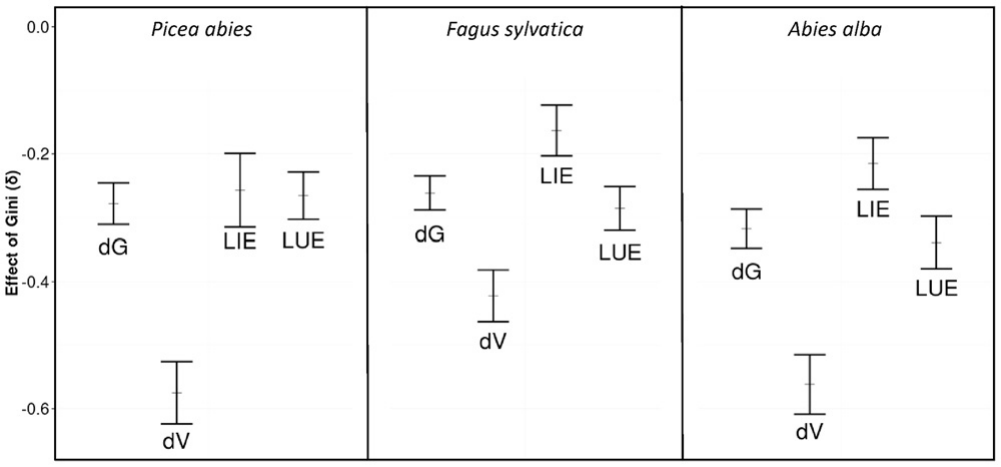
Results from Samsara2 simulations. Production in basal area (dG/dt), production in volume (dV/dt), total light interception (LIE), and light conversion rate (LUE) for *Fagus sylvatica*, *Picea abies* and *Abies alba*. Error bars indicate the confidence interval of δ for each variable represented.

Results based on Samasara2 simulations confirmed the negative effect of tree size inequality on the productivity of pure stands of *Abies alba*, *Fagus sylvatica* and *Picea abies* (Fig 3). The values obtained for parameter δ were consistently lower than those obtained with FNGA data for the three species considered: European beech (δ_FNGA_ = −0.13 and δ_Samsara2_ = −0.26), Norway spruce (δ_FNGA_ = −0.08 and δ_Samsara2_ = −0.28) and silver fir (δ_FNGA_ = −0.09 and δ_Samsara2_ = −0.32). The volume increment was more negatively impacted by tree size inequality than the basal area increment. This difference was less pronounced for European beech (δ_dG_ = −0.26 and δ_dV_ = −0.42) than for the two conifer species (δ_dG_ = −0.28 and δ_dV_ = −0.57 for Norway spruce and δ_dG_ = −0.32 and δ_dV_ = −0.56 for silver fir). Tree size inequality had a negative effect on both LUE and LIE for the three species we studied (Fig 3). More specifically, tree size inequality impacted LUE and LIE with the same magnitude as for Norway spruce (δ_LIE_ = −0.26 and δ_LUE_ = −0.27), whereas it had a more negative effect on LUE than LIE for European beech and silver fir (δ_LIE_ = −0.16 and δ_LUE_ = −0.28 for European beech and δ_LIE_ = −0.21 and δ_LUE_ = −0.34 for silver fir). The negative effect of tree size inequality on productivity appeared related to both a decrease in total light interception and a decrease in the conversion of light into basal area increment and volume.

## Discussion

### A consistent effect of tree size inequality

We found that plots with low levels of tree size inequality were generally more productive than plots with high levels of tree size inequality. This effect was significant for seven out of the ten species studied; it is the first time such result is confirmed on a large set of species. For the three other species (*Quercus pubescens*, *Larix decidua* and *Picea abies*), although not significant, the trend also leaned toward a negative effect of tree size inequality on productivity. These results are consistent with the ones from Cordonnier and Kunstler [13], who showed a negative effect of tree size inequality for two species (*Fagus sylvatica* and *Abies alba*) using a model scaling up individual level growth and competition to the stand scale. Using a density-dependent matrix model for two coniferous species [22] or studying natural conifer stands of western North America [23], Liang *et al*. also found a negative effect of tree-size diversity on respectively diameter growth and recruitment in their model and net basal area growth in natural stands. Our results also extend results from Ryan *et al*. [20] on Eucalyptus plantations in Brazil to more tolerant species. Finally, they are also compatible with the results reported by Long & Shaw [19], who found no effect of tree size inequality on the growth of stands dominated by *Pinus ponderosa* with various combinations of compositional and structural diversity because the productivity of some species appeared to be poorly driven by tree size inequality in our study.

### Reconsidering the role of shade tolerance

Based on the study reported by Cordonnier and Kunstler [13], we hypothesized that the relationship between stand productivity and tree size inequality would be related to species shade tolerance, with more tolerant species less sensitive to asymmetric competition and thus less sensitive to tree size inequality at the stand scale. However, we found no correlations between species shade tolerance indexes and the effect of tree size inequality on production. A potential explanation for this is a positive correlation between species shade tolerance and species ability to intercept light that could cancel out the effect of shade tolerance in monospecific stands. Indeed, although shade-tolerant species might grow more efficiently at low light levels than shade-intolerant species, they could also intercept more light in the upper strata, leading to poor light conditions in the understory. Positive relationships between interception efficiency and shade tolerance have been reported by Niinemets [60]. Shade-intolerant (deciduous or coniferous) species were also shown to transmit more light than shade-tolerant tree species [61], but this result cannot be generalized and seems dependent on species identity. Other species’ characteristics may explain the variations observed in response to tree size inequality between species. For instance, species self-tolerance, a concept introduced by Zeide (1985) [62] for even-aged stands, indicates to what degree a species enforces self-thinning for a given increase in diameter and is related to a species’ lateral canopy expansion ability. Pretzsch [63] showed that low self-tolerance was synonymous with severe intraspecific competition due to high lateral canopy expansion. Such low self-tolerance could partly explain why European beech, a species characterized by both high shade tolerance and high canopy expansion, develops poor stratification in pure dense stands. Lastly, the four species for which we found no or little effect of tree size inequality (*Quercus pubescens*, *Larix decidua*, *Picea abies* and *Pinus halepensis)* are mainly restricted to harsh conditions (high elevations or xeric conditions), where facilitation processes such as water uplift or nursery effect [64] may be more frequent and thus cancel out the importance of size-asymmetric competition.

### Tree size inequality, LUE and LIE

In a simulation that modeled only light competition, we found a negative effect of size asymmetry on productivity on both basal area and volume increment. For the two conifer species, the effect on the volume was almost twice the effect on the basal area, whereas the European beech was less impacted, perhaps due to a difference in crown architecture. Competition for light and asymmetric competition therefore appear crucial to explaining the negative effect of the Gini index in the NGA data. This negative effect of size asymmetry was due to both reduced LUE and reduced LIE in size-structured stands. Both effects were of the same magnitude. To understand the effect of the Gini index, one can consider that among stands of comparable basal area and mean diameter, an increase of the Gini coefficient results in greater diameters between the biggest and smallest trees. Because tree canopy diameter and depth generally reach an asymptote with tree size, numerous studies have shown that LIE also reaches an asymptote with tree size [30,65–68]. A negative effect of the Gini coefficient on LIE can therefore be interpreted as follows: a gain in light interception by bigger trees is unlikely to compensate the loss of interception by smaller trees. A decrease of LUE with tree size inequality appears more surprising. The literature on variation of LUE with tree size is limited; most papers support an increase in LUE as tree size increases [65–69], but a reduction of LUE with size has already been reported at the tree scale [30]. This can be related to the fact that large individuals are mainly in high light conditions where an increase of light only slightly improves their growth, whereas small individuals are in low light conditions where supplementary light has a strong effect on growth. An alternative explanation may also be that large individuals are often older and can suffer from physiological limitations of their LUE due to aging, whereas small trees are usually younger.

A last interesting point emerges from the simulation with Samsara2. The model simulation showed a greater effect of the Gini coefficient index on productivity than the effect observed in NGA data. This could indicate that other competitive mechanisms, less affected by size asymmetry than competition for light, could be important in the natural ecosystem and could explain why the tree size inequality effect appears smaller than the effect predicted using a model based only on light competition.

### Perspectives

The effect of tree size inequality on forest productivity was studied here in monospecifc stands. In mixed-species stands, it is widely believed that there is a potentially positive effect of the differences of species’ shade tolerance within a stand on its productivity [7,70,71]. Most studies demonstrated higher productivity levels in mixtures with stratified canopies (the intolerant species occupying the higher stratum) compared to monocultures [70]. Studying mixtures of *Picea abies* and *Abies alba*, Forrester & Albrecht (2014) [72] showed a complementarity effect associated with increases in both LIE and LUE. Morin et al. (2011) [7] demonstrated a positive effect of species diversity on productivity through an increase in shade tolerance diversity. They also found higher size heterogeneity in more productive forests. The latter results indicate that tree size inequality may have different consequences on ecosystem functioning in pure *vs*. mixed forests, calling for better theoretical and mechanistic understanding of the tree size inequality–productivity relationships in mixed forests. It is important to note that our study does not conclude that high levels of tree size inequality should be avoided in production forests. Other studies based on more dynamical perspectives and integrating management actions proved that high tree size inequality is not incompatible with high wood production and economic returns [44,73]. In other respects, tree size inequality has been shown to be beneficial to several important components of biodiversity of forest ecosystems such as understory vegetation cover and composition, bird diversity and forest regeneration [44,74,75]. The integration of all these components to find an optimal balance between ecosystem services in the long-term is still an avenue of research.

## Supporting Information

S1 Appendix Description of the French National Geographic Agency data and climatic data. (PDF)

S2 Appendix Comparison of different analysis methods: lm *vs*. nls. (PDF)

S3 Appendix P-values from the models for the ten species. (PDF)
