# Supplementary material for: Tree Size Inequality Reduces Forest Productivity: An Analysis Combining Inventory Data for Ten European Species and a Light Competition Model
Source: PLoS One. 2016 Mar 21;11(3):e0151852. doi: 10.1371/journal.pone.0151852 (PMC4801349; doi:10.1371/journal.pone.0151852)
Supplement: S1 Appendix — (PDF) [file pone.0151852.s001.pdf]

S1_Appendix : Description of the French National Geographic Agency data and climatic data

Georges Kunstler

Introduction

This document describes the data from the French National Forest Inventory (NFI) and the climatic data used in Bourdier et al. submitted.

Data description

The French National Forest Inventory comprises a network of temporary plots established on a grid of approximately 1000 x 1000 m. Ten percent of the cells in this grid is sampled each year (we used data from 2006 to 2011). If a particular grid node falls within a forested area, a plot is established (randomly located in a square of 450m around the center of the cell), the soil type is characterized and dendrometric data is measured. Measurements are taken in three concentric circular subplots of different radii, based on circumference at breast height (C_{130}). All trees with $C_{130} > 23.5$ cm, > 70.5 cm and > 117.5 cm were measured within a radius of 6 m, 9 m and 15 m, respectively. For each measured tree, stem circumference, species, status (dead or alive, including only tree that died less than five years ago according to bark and small branches state), and radial increment over five years were recorded. The radial increment was determined from two short cores taken at breast height. Soil properties were analysed using a soil pit of up to 1 m depth located in the center of the plot. One or two soil horizons were distinguished from the soil pit, and depth, texture (based on eight classes) and coarse fragment content were recorded for each horizon. Soil water holding capacity ($SWHC$) was computed based on these three variables, using standard values of water retention for each texture class (following Piedallu et al. 2011).

The following document give details about data formatting and the computation of the basal area at the two dates.

Data downloaded

The data were downloaded from IGN website for each of following year of inventory: 2006, 2007, 2008, 2009, 2010 and 2011. For each year, four files are provided: individual alive trees data, individual dead trees data, ecological data. It was needed to merge data for each year and to homogenize the different variables because the variables and the category of the variables have changed between years (see at the end of the document). In addition we purchased form IGN the exact elevation of the plot (the exact coordinates of plots are not available only the center of the 1x1km cell where the plot is located).

Simplified tree

If the number of trees of a given species and a given size class (C_{130} classes 23.5-70.5, 70.5-117.5, 117.5-164.5, >164.5cm) is greater than 6, the radial increment is measured only on 6 individuals. To compute the basal area on the plot 5-years ago it is needed to estimate the growth of the trees lacking growth measurement (simplified trees). We explored two methods to predict the growth of these individuals.

- We affected to the individual with missing growth data on a plot the average growth rate of the measured individual of the same species and same size class.

- We fitted mixed model of radial increment (dG) as a function of C_{130} ($log(dG) = a_p + b \times log(C_{130})$ with a random intercept drawn for each plot (a_p). We then predicted radial increment for each tree with missing data according to their C_{130} and their plot. The model was fitted in a Hierarchical Bayesian framework using JAGS (Plummer 2003).

We used the prediction of the average growth rate of the measured individual of the same species and same size class because it seemed to have less uncertainty than a modeling method and both approaches provided us with fairly similar results.

Climatic data

For each plots the monthly temperature and total precipitation was taken from a GIS data base at $1km_2$ resolution developed by Bertrand et al. (2011) and Piedallu et al. (2013). The solar radiation accounting for cloudiness cover was also retrieved for each plots from a data base averaged at 1 km_2 resolution (Piedallu & Gegout 2008). The temperature was corrected for the actual elevation of the plot using geospatial correction of the temperature laps rate.

Based on this data we computed the sum of degree days above 5.56 $°C$ ($SGDD$) and a water stress index computed from a monthly water budget (using the model of Bugmann & Cramer 1998) (WB).

- To compute the $SGDD$ we used a spline of the average monthly temperatures.
- The water stress index (WS) is based on the ratio of the actual evapotranspiration over the potential evapotranspiration (the details of the calculation is presented below).

Water budget model

The monthly potential evapotranspiration (PET_m) was computed using the Turc equation (Turc, 1961).

$$PET_m = n \times 0.0133333 \times (Rg_m + 50) \times (t_m/(t_m + 15) \quad (1)$$

with n = number of days of the month, t_m = the monthly temperature and Rg_m = the monthly radiation. The water budget computed monthly soil water content (SWC_m), with initial condition for January SWC_m set as $SWHC$ (SHWC is the maximum soil water content). Then monthly soil water content was iteratively computed using the following equation.

$$SWC_{m+1} = min(SWC_m + Ps_m - AET_m, SWHC) \quad (2)$$

with Ps_m = the infiltrating precipitation, AET_m = the monthly actual evapotranspiration. $AET_m = min(D_m, S_m)$ with $D_m = PET_m$ĂŞPi_m where Pi_m is the the intercepted precipitation. and $S_m = cw * SWC_m/SWHC$ where cw is a parameter denoting the maximum evapotranspiration from a saturated soil under conditions of high demand (as in Bugmann & Cramer 1998 we assume that $cw = 12$ cm/month).

Pi_m and Ps_m are computed as: $Pi_m = min(fi * P_m, PET_m)$ with fi = a parameter denoting the fraction of precipitation that is intercepted and is set at a value of 0.3 following Bugmann & Cramer (1998), and P_m = the monthly precipitation. $Ps_m = P_m - Pi_m$

The water stress index was computed as

$$WS = \frac{\sum_{m=1}^{12} AET_m}{\sum_{m=1}^{12} PET_m} \quad (3)$$

Soil water holding capacity

$SWHC$ was computed following (Piedallu et al. 2011) as

$$SWCHC = (1 - RO) \times (\sum_{i=1}^{n} (1 - \sqrt{SC_i}^3) \times (\theta_i^{2.0} - \theta_i^{4.2}) \times T_i) \quad (4)$$

with n the number of horizons in the soil profile. SC_i is the stone proportional content in horizon i, $\theta_i^2.0$ and $\theta_i^4.2$ are the water content at respectively -100 hPa and -15000hPa matric potential of horizon i (according to Al Majou et al. 2008), T_i is the thickness of the horizon i in millimeters and RO is the proportion of rock outcrop recoded for the plot.

Matching of different years

Alive tree

(1) In 2006 the variable *veget* had either the value 0 no damaged or Z damaged. From 2007 the damaged have been recorded in the variable *acci* with the value 0 for no damage and 1 to 5 for different type of damage. A variable $vege4 with value 0 no damage or 1 damage have been created for all year.

(2) Variable *orir* give the origin of the tree: recruit from seed (1) or from resprouting (0 only in 2005 and 2006 - but 0 for resprout and 2 for resprout from wind thrown tree from 2007 and onward).

(3) Variable $simplif$ show which the tree that were simplified only after 2009.

(4) Variables $sfgui$ $sfgeliv$ $sfpied$ $sfdorge$ $sfcoeur$ were provided only after 2009.

Plot data

(5) The variable *plisi* occurrence of an edge was not recorded in 2006.

(6) The variable *incid* occurrence of a disturbance was not recorded before 2009.

Ecological data

We only used the pedological variables. There was no changes in variables between years for the variables we used (soil description).

References

- Al Majou, H., Bruand, A., Duval, O., (2008) The use of in situ volumetric water content at field capacity to improve the prediction of soil water retention properties. Canadian Journal of Soil Science, **88**, 533-541.
- Bugmann, H. & Cramer, W. (1998) Improving the behaviour of forest gap models along drought gradients. Forest Ecology and Management,**103**, 247-263.
- Bertrand, R., Lenoir, J., Piedallu, C., Riofrio-Dillon, G., de Ruffray, P., Vidal, C., Pierrat, J.C., Gegout, J.C., 2011. Changes in plant community composition lag behind climate warming in lowland forests. Nature, 479, 517-520.

- Kunstler, G., Albert, C.H., Courbaud, B., Lavergne, S., Thuiller, W., Vieilledent, G., Zimmermann, N.E., Coomes, D.A. (2011) Effects of competition on tree radial-growth vary in importance but not in intensity along climatic gradients. Journal of Ecology, **99**, 300â “312.
- Piedallu, C., and Gegout, G. (2008) Efficient Assessment of Topographic Solar Radiation to Improve Plant Distribution Models. Agricultural and Forest Meteorology, **148**, 1696â “1706.
- Piedallu, C., J. C. GÃ©gout, A. Bruand, & Seynave, I. (2011) Mapping Soil Water Holding Capacity over Large Areas to Predict Potential Production of Forest Stands. Geoderma, **160**, 355â “366.
- Piedallu, C., Gegout, J.C., Perez, V., Lebourgeois, F., 2013. Soil water balance performs better than climatic water variables in tree species distribution modelling. Global Ecology and Biogeography, **22**, 478-482.
- Plummer, M. (2003) JAGS: A program for analysis of Bayesian graphical models using Gibbs sampling. In Proceedings of the 3rd International Workshop on Distributed Statistical Computing (DSC 2003). March, pp. 20â “22.
- Turc, L. (1961) Evaluation des besoins en eau dâ irrigation, Ã©vapotranspiration potentielle. Annales Agronomiques, **12**, 13-49.
