# Supplementary material for: Tree Size Inequality Reduces Forest Productivity: An Analysis Combining Inventory Data for Ten European Species and a Light Competition Model
Source: PLoS One. 2016 Mar 21;11(3):e0151852. doi: 10.1371/journal.pone.0151852 (PMC4801349; doi:10.1371/journal.pone.0151852)
Supplement: S2 Appendix — (PDF) [file pone.0151852.s002.pdf]

S2_Appendix : Comparison of different analysis methods: lm vs. nls.

This appendix has been created to compare two different methods we tested to analyse the data from the French National Forest Inventory used in Bourdier et al. submitted.

The two methods tested were:

1. The log transformed version of the model

   $$log(dG) = \alpha_0 + \alpha_1 WB.y + \alpha_2 sgdd + \beta logBA + \gamma QMD7.5 + \delta GCba + \epsilon$$

2. Non-linear model using ‘nls’ in R

   $$dG = e^{\alpha_0+\alpha_1 WB.y+\alpha_2 sgdd} * BA^{\beta} * e^{\gamma QMD7.5+\delta GCba} + \epsilon$$

AIC table showing results from both models

Species	deltaAIC_lm	deltaAIC_nls
PinSyl	0	854.54
QuePet	0	568.3
QueRob	0	386.26
PinPin	0	390.08
FagSyl	0	456.3
QuePub	0	279.53
AbiAlb	0	77.74
PinHal	0	49.44
PicAbi	0	66.4
LarDec	0	93.44

For all species, the linear model using lm on the log transformed scale yields better results than the nls method.
