# Supplementary material for: Tree Size Inequality Reduces Forest Productivity: An Analysis Combining Inventory Data for Ten European Species and a Light Competition Model
Source: PLoS One. 2016 Mar 21;11(3):e0151852. doi: 10.1371/journal.pone.0151852 (PMC4801349; doi:10.1371/journal.pone.0151852)
Supplement: S3 Appendix — (PDF) [file pone.0151852.s003.pdf]

S3_Appendix: P-values from the models for the ten species

This document presents the p-values from the lm models used in Bourdier et al. submitted.

Species	(Intercept)	WB.y	logBA	QMD7.5	GCba	sgdd
PinSyl	1.090e-117	1.322e-09	2.857e-100	2.277e-40	6.343e-03	NA
QuePet	1.133e-131	6.726e-15	2.852e-101	4.133e-79	2.307e-05	NA
QueRob	8.672e-48	1.171e-04	7.422e-87	1.981e-103	2.428e-08	5.034e-02
PinPin	7.276e-10	1.436e-15	2.684e-117	5.361e-128	2.206e-17	8.589e-06
FagSyl	3.975e-52	6.598e-08	4.435e-50	6.082e-72	1.741e-06	3.212e-12
QuePub	1.174e-66	5.516e-08	4.381e-99	9.112e-37	6.565e-01	1.226e-01
AbiAlb	3.441e-21	8.966e-05	5.256e-28	1.273e-32	3.163e-02	1.163e-06
PinHal	2.384e-31	NA	2.236e-23	3.010e-15	4.748e-02	NA
PicAbi	6.341e-20	1.659e-04	2.169e-27	3.796e-22	8.743e-02	5.205e-05
LarDec	1.185e-21	6.998e-02	1.511e-12	3.983e-14	6.934e-01	NA
